# Dynamic Interaction of cBid with Detergents, Liposomes and Mitochondria

**DOI:** 10.1371/journal.pone.0035910

**Published:** 2012-04-23

**Authors:** Stephanie Bleicken, Ana J. García-Sáez, Elena Conte, Enrica Bordignon

**Affiliations:** 1 Department of Membrane Biochemistry, Max Planck Institute for Biochemistry, Martinsried, Germany; 2 Department of Membrane Biophysics, BIOQUANT, Max Planck Institute for Intelligent Systems and German Cancer Research Center, Heidelberg, Germany; 3 Centro di Studio sui Mitocondri e Metabolismo Energetico (CNR) c/o Dipartimento di Biochimica e Biologia Molecolare, Università di Bari, Bari, Italy; 4 Laboratory of Physical Chemistry, ETH Zurich, Zurich, Switzerland; The University of Texas MD Anderson Cancer Center, United States of America

## Abstract

The BH3-only protein Bid plays a key role in the induction of mitochondrial apoptosis, but its mechanism of action is still not completely understood. Here we studied the two main activation events of Bid: Caspase-8 cleavage and interaction with the membrane bilayer. We found a striking reversible behaviour of the dissociation-association events between the Bid fragments p15 and p7. Caspase-8 cleavage does not induce *per se* separation of the two Bid fragments, which remain in a stable complex resembling the full length Bid. Detergents trigger a complete dissociation, which can be fully reversed by detergent removal in a range of protein concentrations from 100 µM down to 500 nM. Incubation of cBid with cardiolipin-containing liposomes leads to partial dissociation of the complex. Only p15 (tBid) fragments are found at the membrane, while p7 shows no tendency to interact with the bilayer, but complete removal of p7 strongly increases the propensity of tBid to become membrane-associated. Despite the striking structural similarities of inactive Bid and Bax, Bid does not form oligomers and reacts differently in the presence of detergents and membranes, highlighting clear differences in the modes of action of the two proteins. The partial dissociation of cBid triggered by the membrane is suggested to depend on the strong and specific interaction between p15 and p7. The reversible disassembly and re-assembly of the cBid molecules at the membrane was as well proven by EPR using spin labeled cBid in the presence of isolated mitochondria. The observed dynamic dissociation of the two Bid fragments could allow the assistance to the pore-forming Bax to occur repeatedly and may explain the proposed “hit-and-run" mode of action of Bid at the bilayer.

## Introduction

Bcl-2 proteins are key regulators in the mitochondrial dependent cell death pathway [1], [2]. The protein family can be divided into three subgroups: the anti-apoptotic Bcl-2-like proteins (e.g. Bcl-xL), the pro-apoptotic multi-domain proteins (e.g. Bax or Bak) and the pro-apoptotic BH3-only proteins (e.g. Bid or Bim). The latter ones can be further subdivided in activators and sensitizers, depending on their mode of action (recently reviewed in [3]). Bcl-2 proteins are crucial in cancer development and therefore important targets in drug development (e.g. [4]).

The activator BH3-only protein Bid links the death receptor pathway to the permeabilization of the outer mitochondrial membrane [5], [6]. In non-apoptotic cells Bid is present in the cytoplasm, where it can be cleaved by Caspase-8 [7]. The resulting cleaved protein is called cBid and contains two protein fragments p7 and p15 (tBid). Only after cleavage, Bid is able to trigger apoptosis [8], [9], [10], [11], [12]. *In vitro*, both cBid and tBid variants are shown to induce Bax activation [13], [14]. tBid is proposed to be the active fragment and p7 to act like an “inhibitor" (e.g. [15], [16]). However, it is not completely understood whether tBid is the only active factor in the living cell or also cBid is active. If tBid is the only active form, p7 needs to be removed. *In vivo*, proteasomal degradation of p7 was detected [17], offering an elegant mechanism of p15 release, but as p15 was also shown to be degraded [17], [18], the mechanism was not fully clarified. Even *in vitro* it is contradictorily discussed what happens to cBid when membranes and Bax are present. We previously found only a minority of cBid bound to the membrane [13], while others stated that it is the majority [14].

**Figure 1:**
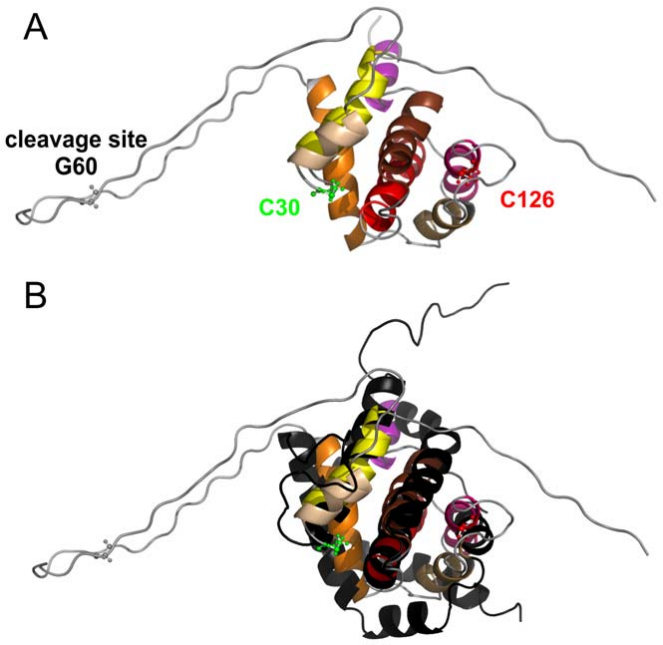
Structural similarities between mouse Bid and human Bax. A. NMR structure of mouse Bid (PDB 1DDB) with the two natural cysteines spin labeled in this study (C126 in p15 and C30 in p7) and the cleavage site in ball and stick models. B. Superposition of mouse Bid (colored) and human Bax (black, PDB 1F16). Color code for alpha helices in mouse Bid: 1, light yellow; 1/2, dark yellow; 2, orange; 3, gold; 4, pink; 5, red; 6, brown; 7, violet.

The structures of soluble inactive conformations of several Bcl-2 proteins, including Bid, were solved by NMR (e.g. [19], [20], [21], [22], [23]). NMR studies on human and mouse Bid [22], [23] found that it has a globular fold very similar to Bax and Bcl-xL (Fig. 1). This is in contrast to other BH3-only proteins which are reported to be mainly unfolded [24]. Bax and Bcl-xL are composed of nine alpha helices (α1 to α9) with α2 representing the BH3 domain, α5/α6 the hydrophobic hairpin, and α9 being important for membrane insertion. Bid lacks α8 and α9 and has an additional short helix (α1/2) between α1 and α2 (Fig. 1). The Caspase-8 cleavage in Bid takes place in a loop before the BH3 domain in helix 2 (reviewed in [7]). Notably, we use here a helix nomenclature which refers to the Bcl-2 protein fold. Thus, we name α1/2 the short second helix, which is unique for Bid, while others may refer to it as helix 2, which changes the nomenclature of all following helices.

Although Bcl-2 proteins have been intensively studied for two decades now, major questions concerning their structure, function and interaction are still unanswered. Especially, the structures of the active membrane-inserted or membrane-associated conformations are poorly understood and only local information is available.

Evidence for the existence of a transmembrane motif [25], [26] and of a BH3-BH3 interaction in the membrane-inserted form of Bax and Bak [13], [27], [28] starts to accumulate, unveiling first structural details of the oligomeric membrane-bound form of the multi-domain pro-apoptotic proteins.

In the case of Bid, an NMR study showed that after cleavage the protein does not undergo conformational changes at millimolar concentrations, and suggested that at lower concentration spontaneous dissociation may occur [23]. After membrane reconstitution, tBid was shown by site directed spin labeling EPR to partially unfold and become membrane-associated, allowing exposure of the BH3 domain [29]. Studies on human Bid demonstrated that tBid adopts a unique helical fold in membrane environments, binding to the membrane without insertion of its helices [30].

Bcl-xL is also described to insert into membranes and to interact with Bid and Bax (e.g. [26], [31], [32]). Moreover, Bcl-xL is believed to be a kind of inhibitor of Bax-induced membrane permeabilization (e.g. [31]), but it was also shown that it can permeabilize membranes by itself [32], [33]. Thus the interplay of Bid, Bax and Bcl-xL is far from being understood.

This study focuses on the properties of the BH3-only protein Bid in its three forms: full length Bid (FL-Bid), cBid and tBid in aqueous solution and in the presence of detergents or liposomes. To address the dynamic properties of cBid in a physiological environment by EPR, we used for the first time site directed spin labeling in the presence of isolated mitochondria from rat liver. No reconstitution attempts were performed, but the different forms of the protein were allowed to interact spontaneously with the different environments. With respect to Bax, Bid responds differently to detergents and membranes highlighting remarkable differences in the modes of action of the two proteins despite the striking similarities in their inactive conformations.

Our data confirm the very stable interaction between the Caspase-8 cleavage products p7 and p15 found by NMR [23]. Additionally, we show that this tight interaction holds the fragments together even at submicromolar protein concentrations, proving that it is not an artefact due to the high protein concentration, but an intrinsic property of cBid. This fact demonstrates that the separation of both fragments needs an extra trigger besides the cleavage (e.g. detergents or lipids). Surprisingly, we found that fragment dissociation is fully reversible, highlighting a unique feature of cBid to reversibly switch between two conformations: an assembled and soluble p15-7 complex and the two separated fragments from which p15 (tBid) is membrane associated. The reversibility of the assembly-disassembly, which could also be shown in the presence of mitochondria provides a possible molecular basis for the “hit-and-run" model of Bid-induced Bax activation.

## Results

### Bid does not oligomerize

**Figure 2:**
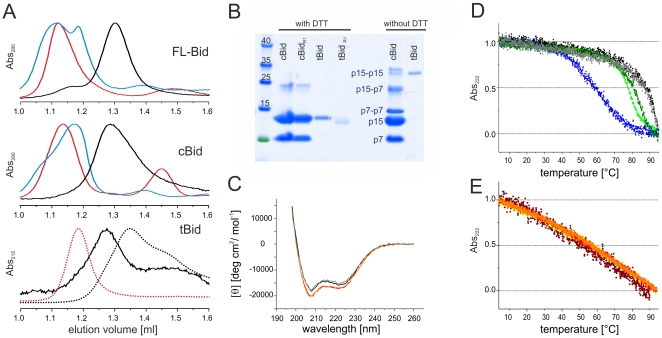
Oligomerization and structural stability of Bid. A. SEC chromatograms (Superdex 75 column) of FL-Bid, cBid and tBid. Black curves, soluble proteins; dark cyan, 2% OG; red, 1% DDM. For tBid the elution profile in the presence of DTT is also shown (dotted lines). B. SDS PAGE of cBid and tBid (before and after spin labeling) in the presence and absence of DTT. C. CD spectra of soluble and detergent-incubated (1% DDM) FL-Bid (black and red, respectively); soluble and detergent-incubated (1% DDM) cBid (gray and orange, respectively). D. Normalized melting curves of the Bid variants. Black and gray, soluble FL-Bid and FL-Bid_R1_, respectively; light and dark green, soluble cBid and cBid_R1_, respectively; blue, soluble tBid. E. Normalized melting curves of the Bid variants in the presence of 1% DDM. Red, FL-Bid; orange and yellow, cBid and cBid_R1_, respectively; brown, tBid.

To characterize the propensity of mouse FL-Bid, cBid and tBid to form oligomers we used SEC (size exclusion chromatography) combined with SDS PAGE in the presence and absence of the reducing agent DTT (Fig. 2). All Bid variants used in this work were shown before to induce Bax activation [13]. As the the nitroxide spin label used for the site directed spin labeling EPR experiments shown later in this work is very sensitive to reducing agents, all EPR experiments were necessarily performed in the absence of DTT. In the labeled proteins all cysteines are covalently bound to the nitroxide label, thus they are not prone to disulfide linkage even in the absence of DTT. However, as we additionally used unlabeled Bid variants in some experiments, we checked that no artificial protein oligomers due to cysteine linkage were present without DTT.

FL-Bid and cBid eluted as 20 kDa monomers at 1.3 ml without DTT in SEC (Fig. 2A, black lines). Addition of DTT or spin labeling did not affect the elution peaks (data not shown). SDS PAGE on samples with DTT revealed no sign of dimerization in cBid. In the absence of DTT only traces of p15-p7, p15-p15 and p7-p7 disulfide-linked dimers were detected in the cBid sample (Fig. 2B). On the contrary, tBid was found by SEC to be exclusively dimeric in the absence of DTT (black line in Fig. 2A, bottom panel), and mainly monomeric in the presence of DTT (black dotted line in Fig. 2A, bottom panel), indicating the strong tendency of the surface exposed C126 cysteines (unique cysteine in tBid) to form a disulfide bridge. The tBid dimer is an artefact induced by the non-reducing *in vitro* conditions, and it is probably not relevant *in vivo*. The tBid dimer has possibly a different membrane binding activity with respect to the monomer, thus only the spin labeled tBid, which is unable to form disulfide bridges, was further used in this study.

In the presence of detergents FL-Bid and cBid eluted at around 1.1 ml (1% DDM (β-dodecyl-D-maltoside), red and 2% OG (octyl-glucoside), dark cyan in Fig. 2A). The elution volume is consistent with a soluble protein of about 50 kDa (protein ovalbumin - 43 kDa - eluted at 1.16 ml) and this shift likely represents the monomeric form of the protein interacting with the detergent micelle. It is worth noting that Bax eluted comparable to a 440 kDa protein under similar conditions [13]. Removal of DTT had only slight effects on the elution volumes of FL-Bid and cBid, in detergents (not shown). tBid was only analyzed in presence of detergents and DTT and eluted at about 1.2 ml (Fig. 2A).

In summary, SEC demonstrated that tBid is monomeric *in vitro* only in the presence of reducing agents. FL-Bid and cBid interact with the detergent micelles, but do not tend to form oligomers, which is in contrast to observations on Bax [13]. The absence of detergent-induced oligomerization does not indicate a lack of conformational changes in Bid, which will be addressed in the following paragraphs.

### Secondary structure and stability of Bid

According to literature, human and mouse FL-Bid, cBid, and tBid are mainly composed of α-helices [22], [30]. We performed CD spectra on the mouse analogs to confirm the correct protein folding (Fig. 2C, Fig. S1). The CD data confirmed for FL-Bid and cBid a α-helical content of 54–55% and for tBid a predominant α-helical content as well (Fig. S1). Moreover, we found that addition of detergents did not change the overall secondary structure (Fig. 2C).

The stability of all Bid variants was also investigated by thermal denaturation. The CD melting curves showed a sigmoidal behaviour (Fig. 2D). Melting was not completely reversible (not shown). FL-Bid was the most stable derivative with a melting point of 87–90°C (black). Cleavage reduced the melting temperature to 77°C, indicating a decrease in protein stability (light green). tBid was the least stable with a melting point of ∼60°C (blue), demonstrating that p7 is relevant for its stability.

The spin label covalently bound to the natural cysteines had no effect on FL-Bid (grey), and only a slight stabilizing effect was observed on cBid (Fig. 2D, dark green), which might be due to the presence of the spin label itself, or to the removal of p15-p15 and p7-p7 dimers otherwise present in the unlabeled protein sample (as detected by SDS PAGE without reducing agents, Fig. 2B). However, in the temperature range of the EPR experiments (20–37°C) there are no differences between unlabeled and spin labeled proteins.

After detergent addition, the melting curves of all three Bid derivatives changed to an almost identical non-sigmoidal form (Fig. 2E) and the melting was found to be fully reversible (not shown). Thus, addition of detergent changed the thermal stability of Bid. Interestingly, a similar melting behavior in the presence of detergent was also found for Bax [34].

In summary, the CD data showed that the secondary structure of Bid is not affected by Caspase-8 cleavage, removal of p7 or presence of detergents, whereas protein stability is affected by all three factors with caspase cleavage having only a slight effect. Spin labeling is shown to be tolerated by Bid without structural rearrangements. The changes in the melting behavior after addition of detergents clearly show that the detergent affects Bid, likely inducing a conformational change.

### Caspase-8 cleavage primes cBid for dissociation. Detergents separate the cBid fragments

Mouse Bid has two endogenous cysteines: C30 (located directly after α1, in p7) and C126 (at the beginning of α4, in p15, see Fig. 1). Both were spin labeled with MTSSL (methanethiosulfonate spin label) to detect conformational changes by EPR induced by Caspase-8 cleavage and detergents or membranes. For simplicity, the doubly spin labeled full length protein will be named FL-Bid_R1_, the cleaved version containing two spin labels cBid_R1_ and the singly spin labeled p15 fragment tBid_R1_ (R1 symbolizes the attached MTSSL). After cleavage each Bid fragment contains one spin label (Fig. 1). Measuring the distance between the two labels by pulse EPR will give direct information on the dissociation of the polypeptides. Additionally, continuous wave (cw) spectra of all Bid variants were detected to monitor the overall motion of the two labels, thus secondary and tertiary constraints, encoded in the EPR line shape.

**Figure 3:**
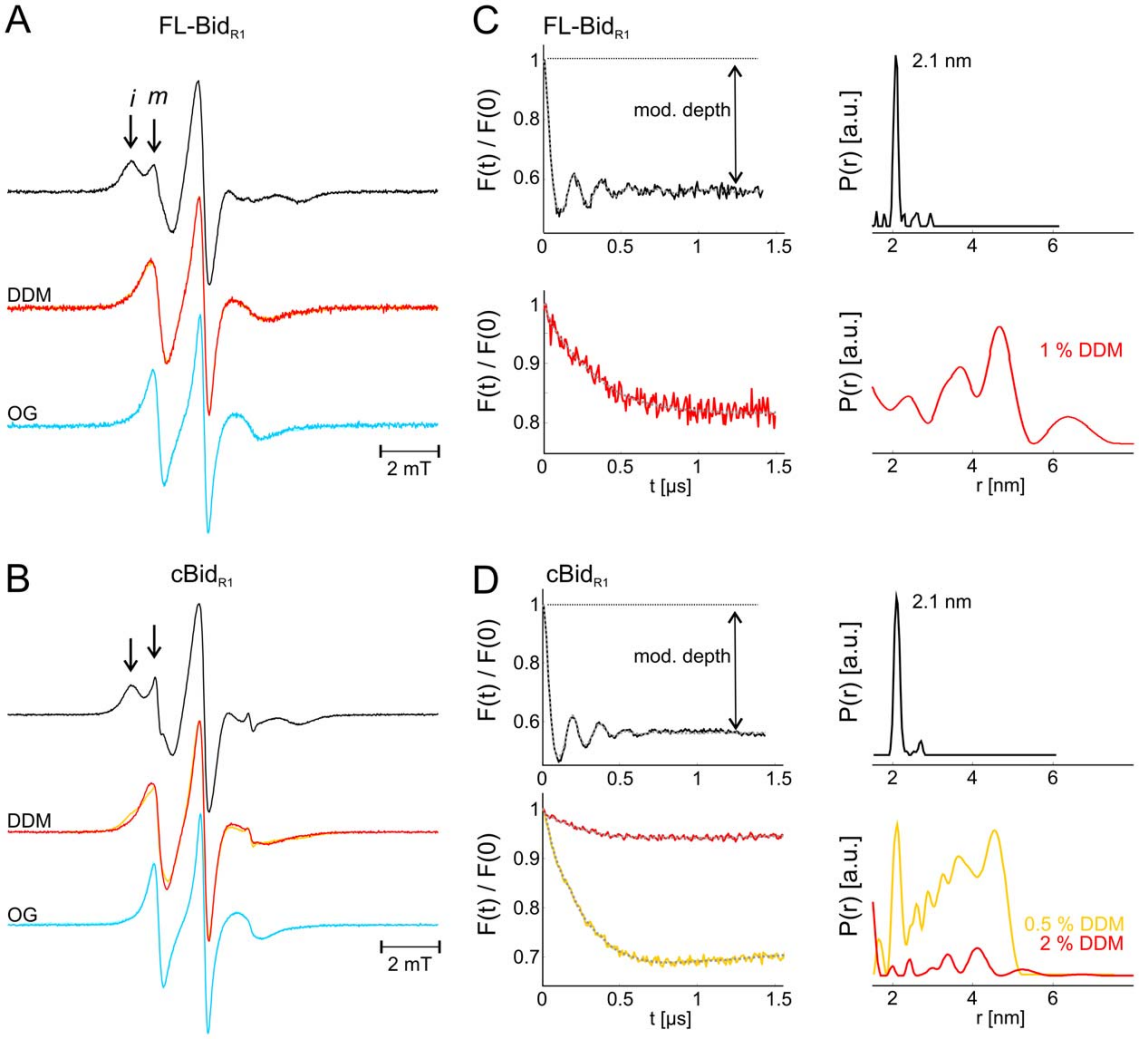
Effect of detergent on Bid structure and dynamics. Room temperature continuous wave EPR spectra of FL-Bid_R1_ (A) and cBid_R1_ (B) in the absence (black) and presence of 0.5 (yellow) and 2% DDM (red) and 1 (cyan) and 2% OG (dark cyan). Arrows highlight the immobile (i) and mobile (m) spectral components. C, D. Normalized form factors F(t) and distance distributions obtained by fit of the DEER traces with the software DeerAnalysis2010 in the absence and presence of detergents (color code as in panel A). Protein concentrations 40 µM.

The cw EPR spectrum of FL-Bid_R1_ showed clearly two spectral components, representative of two spin label populations with a different degree of mobility (Fig. 3A, black spectrum and Fig. S2). The more mobile (m) spin label population is characterized by narrower EPR lines, and the one more restricted in motion (i) has broader EPR lines (see arrows in Fig. 3A). The two spectral fractions however cannot be directly assigned to two distinct dynamics of the two labeled sites, but rather their complex anisotropic motion, as the spectrum of the singly labeled FL-Bid_C126R1_ (having only one cysteine at position 126) also showed two spectral components (Fig. S2).

Interestingly, cleavage by Caspase-8 led only to a minor increase in mobility in the spectra (Fig. 3B, black spectrum), which was also observed in the singly labeled Bid mutant (Fig. S3).

After incubation with increasing concentrations of DDM and OG, the spectra of Bid_R1_ and cBid_R1_ showed a distinct increase in the spin labels mobility (Fig. 3 A, B). The same effects were observed on the singly spin labeled mutants (Fig. S3).

To characterize the extent of cBid fragmentation the distance between the two labels at positions 126 and 30 was measured by Double Electron Electron Resonance (DEER, also known as PELDOR) in FL-Bid_R1_ and cBid_R1_ before and after addition of detergents (Fig. 3 C, D). In absence of detergent the same narrow distance distribution centered at 2.1 nm was measured between the two spin labeled sites in FL-Bid and in cBid. This indicates that the conformation of Bid is preserved after cleavage. Moreover, the invariant modulation depth confirms that all complexes are associated. The interspin distance detected correlates with the 2 nm Cα-Cα average distance between the two residues in the NMR structural models [23].

Upon detergent addition, the two labels moved apart in FL-Bid showing a broad range of distances from 1.5 to 6 nm (Fig. 3C, red traces). This effect can be explained by a dynamic exposure of the protein hydrophobic core. In the case of cBid_R1_ low DDM concentration (0.5%) induced a partial displacement of the two polypeptides (Fig. 3D, orange traces). Addition of higher detergent concentrations (2% DDM) induced an almost complete disassembly of the two polypeptides (Fig. 3D, red traces). The decrease in modulation depth from 0.45 in the absence of detergent (value which describe a 100% population of interacting polypeptides) to 0.06 in the presence of 2% DDM, indicates that only 10% of the fragments are still interacting under these conditions. These results show that the interaction between p15 and p7 is rather strong and complete disassembly following Caspase-8 cleavage only occurs in the presence of high detergent concentrations.

It is worth noting that Bax acts very differently in presence of detergents: it forms high order oligomers with defined inter-monomer distances as detected by EPR [13].

### Reversible dissociation of p15 (tBid) and p7

**Figure 4:**
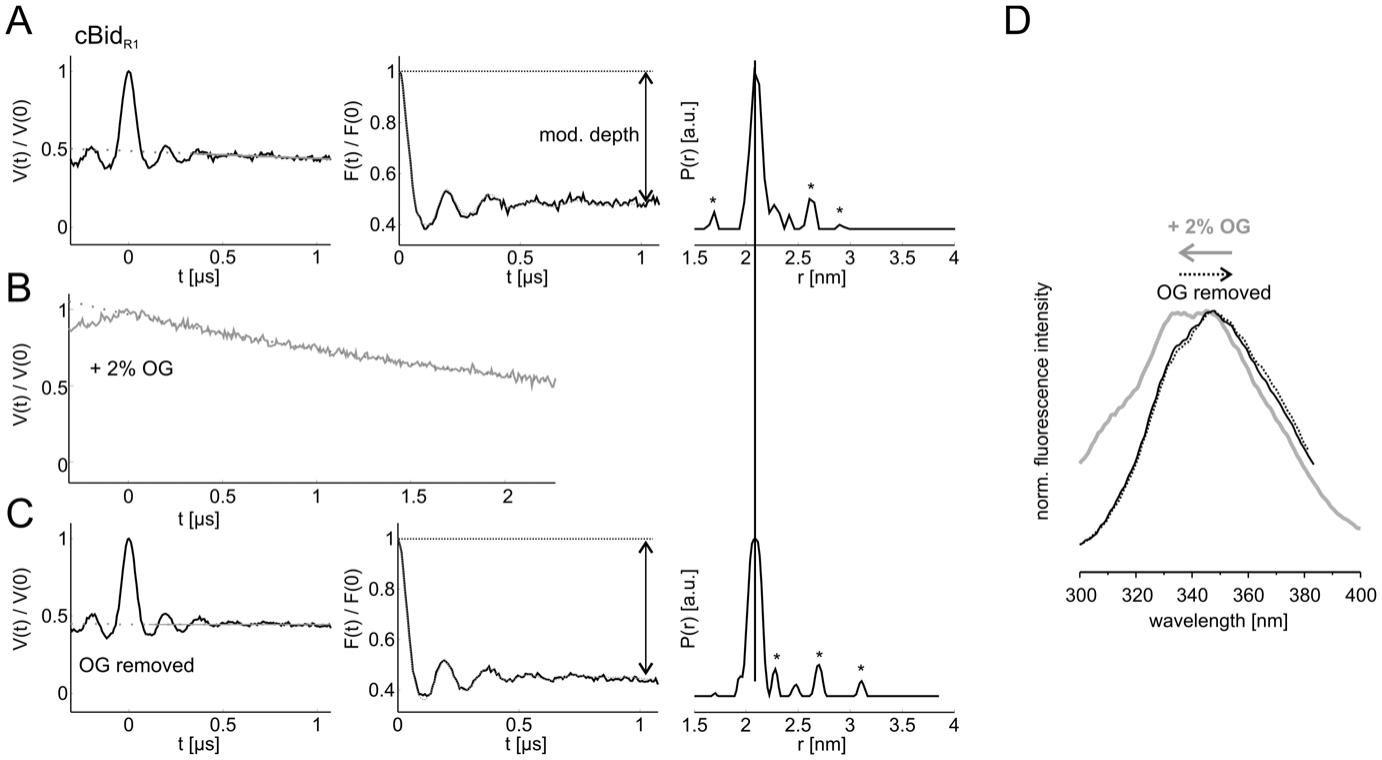
Reversibility of the cBid dissociation at different protein concentrations. (A) Normalized DEER traces V(t), normalized form factors F(t) and distance distributions obtained with the software DeerAnalysis2010 of cBid_R1_ in the absence of detergent (A), in the presence of 2% OG (B, only V(t) is presented), and after removal of detergent by extensive dialysis (C). Asterisks denote noise-related artefacts in the distance distribution. Protein concentration 180 µM. (D) Tryptophan and tyrosine (Y47, W48, Y185) fluorescence spectra of cBid_R1_ detected after excitation at 280 nm in the absence of detergent (black), in the presence of 2% OG (gray), and after removal of detergent by extensive dialysis (dotted black). Protein concentration 0.5 µM.

The DEER traces of cBid (180 µM concentration) before and after addition of 2% OG are shown in Fig. 4A, B. The detergent is shown to induce complete fragments dissociation (Fig. 4B). Removal of detergent by extensive dialysis resulted in complete reassembly of the two fragments and reappearance of the initial narrow 2.1 nm distance distribution with the same modulation depth in the DEER trace, indicating that all complexes reassembled (Fig. 4C). No evidence for distances arising from newly formed p7-p7 or p15-p15 dimers was obtained. The reversibility of the disassembly induced by OG could also be detected at protein concentrations two to three orders of magnitude smaller (500 nM) by fluorescence spectroscopy. The fluorescence spectra obtained upon excitation at 280 nm characteristic of the tryptophanes and tyrosines in cBid (Y47, W48, Y185) show a distinct shift towards shorter wavelength upon OG addition, which can be fully reversed by detergent removal (Fig. 4D).

The reversibility of the polypeptides disassembly down to submicromolar concentrations proves that the strong and specific interaction between p7 and p15 is a characteristic feature of cBid and not an artefact induced by high protein concentrations. Interestingly, the same attempts to show reversibility for detergent-induced Bax oligomerization were not successful, and mostly resulted in protein precipitation (not shown).

### cBid and tBid: different interactions with liposomes

**Figure 5:**
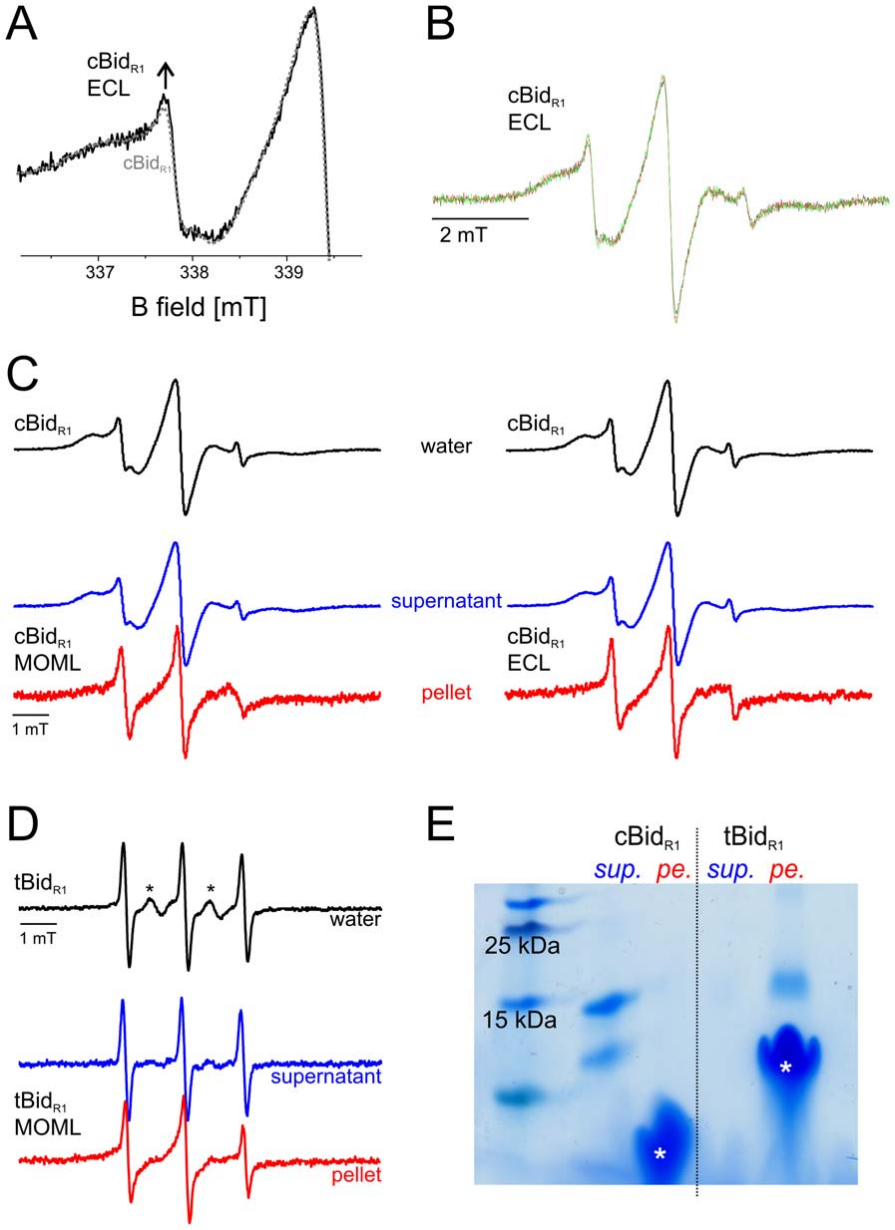
Bid-membrane interactions. A. Low field region of the EPR spectra of cBid_R1_ detected at 37°C in buffer (protein concentration 40 µM, dotted grey) and after 2 minutes incubation (black) of 3.5 µL cBid_R1_ (40 µM) with 4.5 µL ECL liposomes (lipid concentration 4.5 mg/ml). The arrow highlights the small increase in spin label mobility. B. Spectra of cBid_R1_ in the presence of ECL liposomes after 2 minutes (black), 3 h (red) and 16 h (green) incubation. C. Intensity normalized spectra of cBid_R1_ in water (black). Spectra detected in the supernatant fraction (blue) and in the washed pellet fraction (red) after 3 h incubation of 50 µL cBid_R1_ (110 µM) with 50 µL ECL or MOML liposomes (20 mg/ml lipids) at 37°C. The same results were reproduced with 4 times higher lipid to protein ratio (not shown). D. EPR spectrum of tBid_R1_ in water (black). Asterisks denote a small fraction of residual unbound MTSSL biradical in solution. Spectra detected in the supernatant fraction (blue) and in the washed pellet fraction (red) after 3 h incubation of 20 µL tBid_R1_ (50 µM) with with 50 µL MOML liposomes (20 mg/ml lipids). The spectrum in the supernatant is assigned to the residual MTSSL free in solution. E. SDS PAGE of cBid_R1_ and tBid_R1_ in the supernatant fraction (sup.) and in the washed pellet fraction (pe.). The left lane shows the reference molecular weights, asterisks denote the liposome bands. Silver staining of this gel showed some cBid in the pellet fraction as well as some tBid in the supernatant fraction (data not shown).

*In vivo*, Bid needs to translocate from the cytosol to the outer mitochondrial membrane to be able to activate Bax. Thus, we investigated by EPR to what extent the stable interface holding the two Bid fragments together can be destabilized by interaction with the membrane bilayer. To address the cBid dissociation in the presence of membranes we performed experiments with liposomes formed with ECL (*E.coli* polar lipid extracts, containing 9.8% cardiolipin) or a lipid mixture (MOML) mimicking the composition of the outer mitochondrial membrane [14] (1 mg lipid mix contains 0.46 mg egg phosphatidylcholine, 0.25 mg egg phosphatidylethanolamine, 0.11 mg bovine liver phophatidylinositol, 0.10 mg 18∶1 phosphatidylserine, and 0.08 mg cardiolipin). Immediately after addition of ECL liposomes at 37°C a slight increase in mobility was observed for cBid_R1_ (Fig. 5A). Prolonged incubation with liposomes (up to 16 h at 37°C) did not further modify the EPR spectrum, as clearly seen from the two selected spectra detected after 3 and 16 h of incubation (Fig. 5B).

To analyze the minor spectral changes in more detail, the membrane was separated from the soluble fraction by centrifugation and the membrane pellet was washed once to remove unbound cBid molecules and free spin label, possibly released during the incubation time. The EPR spectra showed that the majority of cBid molecules stays in the supernatant fraction while a minor protein fraction is membrane-associated (Fig. 5C). ECL or MOML mixtures showed exactly the same effects. The proteins at the membrane showed a relevant increase in mobility, similar to what was observed upon detergent addition. The SDS PAGE performed on the EPR samples confirmed that most of cBid was in the supernatant, in fact the amount of cBid in the pellet fraction was below the detection limit (Fig. 5E). In our previous work, cBid was mixed with ECL liposomes and the SDS PAGE also showed the majority of cBid in the supernatant, while only a faint band of p15 was found in the membrane fraction, in line with the new EPR data [13].

The same experiments were performed with tBid carrying a single spin label side chain at position 126. The spectrum of tBid in solution shows a remarkably more mobile spin labeled side chain at position 126 than in the cBid (Fig. 5D), however the spectral analysis is complicated by the presence of a fraction of residual free label which could not be removed due to the tendency of tBid to aggregate during the washing steps necessary to eliminate the unbound label. After incubation of tBid with membranes and separation of supernatant and membrane fraction, the spectrum of the supernatant showed basically the features of the free MTSSL (Fig. 5D) present in the sample. The spectrum of the membrane inserted tBid showed a highly dynamic spin label at position 126, in agreement with the spectra obtained for the membrane fraction of cBid_R1_. The EPR samples were analyzed by SDS PAGE, which confirmed that most tBid was in the membrane fraction (Fig. 5E). Our results agree with the findings from several groups showing that tBid inserts spontaneously into membranes containing cardiolipin [14], [32]. The EPR data clearly show that when p7 is present, tBid does not completely insert into membranes.

**Figure 6:**
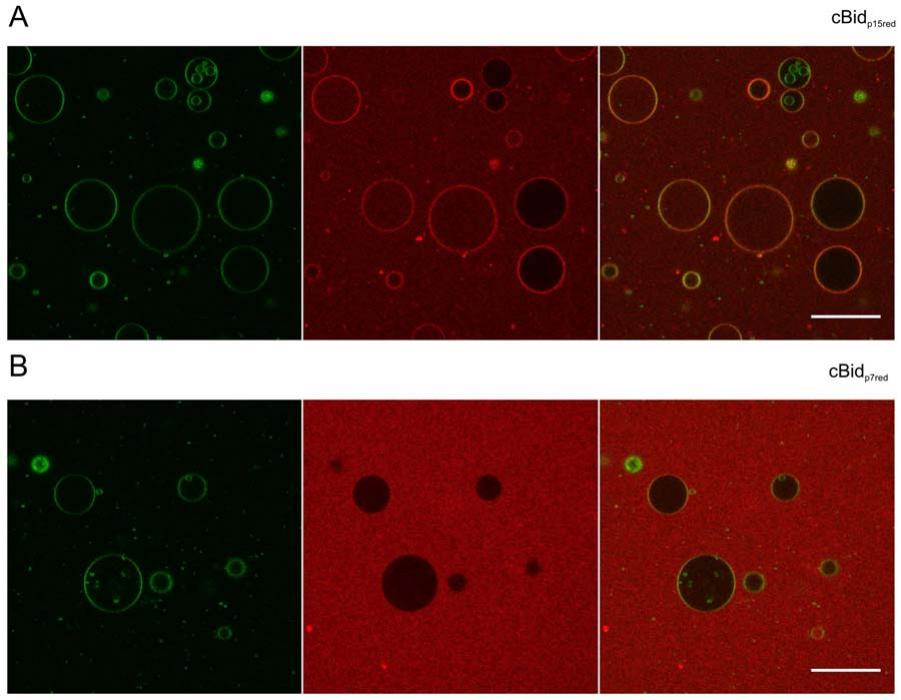
Binding of p15 and p7 to GUVs. cBid_p15red (C30S)_ and cBid_p7red (126S)_ are shown in (A) and (B), respectively. GUVs composed of 80% phosphatidylcholine (egg), 20% cardiolipin (bovine heart) and <0.05%. DiO (Invitrogen) was incubated with 25 nM of the Alexa 633 labeled cBid variant. DiO is shown in the first panel (green) and the Alexa 633 labeled cBid variant in the second (red). The merge of the red and the green channel is shown in the third panel. The bar indicates 50 µm. Pictures were taken after 1 h. Due to better visualization the brightness in the red channel was increase for cBid_p7red (126S)_. Notably, due to the fragility of the GUVs, 10–20% of the total GUVs are permeabilized even in absence of any protein.

To further verify that p15 is the only part of the protein interacting with the membrane and that cBid is not as lipophilic as tBid even at lower protein concentrations, single cysteine cBid variants in which either p7 (Bid-C126S mutant) or p15 (Bid-C30S mutant) were labeled with an alexa 633 dye (cBid_p7red_ and cBid_p15red_) and separately mixed with GUV (giant unilamellar vesicles composed of 80% egg-PC and 20% cardiolipin). In line with the EPR data, the cBid_p15red_ showed that p15 partially co-localizes with the membrane (Fig. 6A) and that the majority of cBid_p15red_ remains in solution, as indicated by the clear alexa 633 background outside the GUVs (Fig. 6A). Performing the same experiment with cBid_p7red_ revealed that p7 remains in the soluble fraction (Fig. 6B). In summary, the presence of liposomes leads only to a partial dissociation of the p15/p7 complex, while the majority of molecules stayed associated in the soluble fraction. ECL and MOM liposomes were both equally suited for experiments with cBid. tBid in contrast to cBid was found mainly in the membrane fraction. The spectral features of the spin label attached to C126 in tBid or to the membrane-bound p15 fragment originating from cBid are similar. Only the p15 fragment is binding to membranes, while p7 stays in solution.

### Reversible cBid binding to isolated mitochondria

EPR experiments on spin labeled proteins in cells or organelles are difficult to perform as the nitroxide radical is extremely sensitive to reducing agents. Only recently, first attempts to overcome these problems have been published [35], [36].

**Figure 7:**
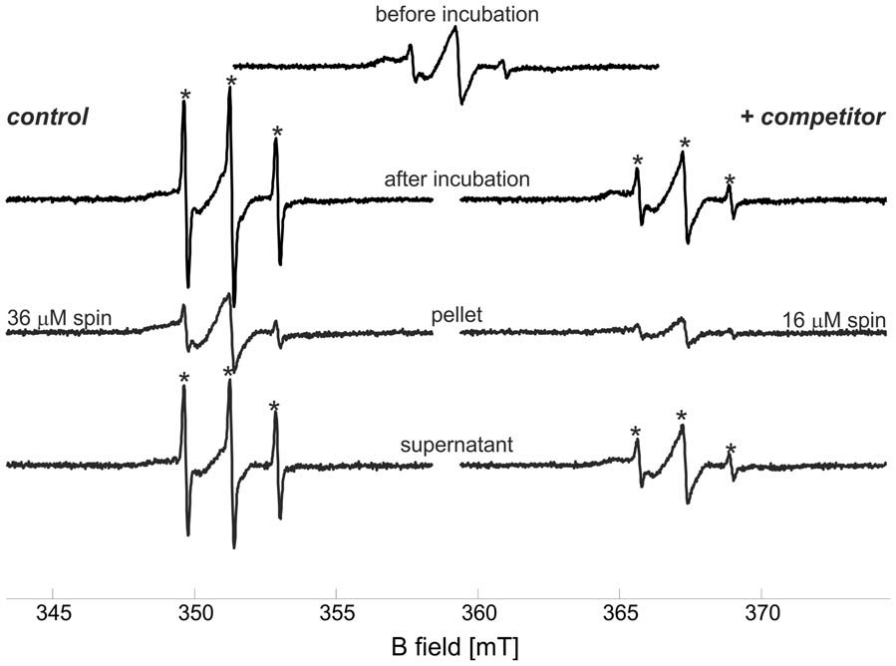
Exchange of Bid molecules at the mitochondrial membrane. Room temperature continuous wave EPR spectra of cBid_R1_ in the presence of isolated mitochondria (1.6 mg/ml lipid concentration). The central upper spectrum of cBid_R1_ is obtained immediately after addition of mitochondria (60 µl sample, 20 µM protein concentration, calculated spin concentration 42 µM). The left column shows the control spectrum after 1 h incubation at 37°C (addition of 20 µl buffer after the first 20 min incubation, spin concentration 32 µM); the spectrum of the pellet resuspended in 10 µl buffer (calculated spin concentration 36 µM) and the spectrum in the supernatant (calculated spin concentration 21 µM). The right column shows the analogous spectra obtained after addition of 14-fold protein excess of competitor unlabeled cBid (addition of 20 µl of cBid 860 µM after the first 20 min incubation, spin concentration 30 µM). The spectrum in the pellet (spin concentration 16 µM) reveals the reduced amount of spin labeled protein at the membrane in the presence of competitor unlabeled protein. In the supernatant the spin concentration was found to be 28 µM.

To study the dynamics of cBid membrane binding under more physiological conditions, we achieved to perform for the first time EPR experiment with spin labeled cBid incubated with isolated mitochondria from rat liver. Addition of spin labeled cBid to isolated mitochondria (1.6 mg/ml lipid concentration) was found not to decrease the intensity of the EPR spectrum, indicating that no reducing agents were released in the buffer (Fig. 7, upper central spectrum). The isolated mitochondria were first incubated for 20 min at 37°C with cBid_R1_. After this first incubation period, the sample was divided into two identical aliquots. A 14-fold molar excess of unlabeled cBid was added to one aliquot, while the same volume of buffer was added to the second as a control. Both samples were further incubated for one hour at 37°C. After incubation, the spectra in the control sample showed a reproducible mobile component, possibly due to release of free spin label (about 15% of the overall spin concentration, highlighted by asterisks in Fig. 7), which was decreased in the presence of unlabeled competitor protein. The slow release of reducing agents from the mitochondria could induce the observed spin label release due to the reduction of the disulfide bond. This effect is more pronounced in the control sample as there are no competing unlabeled cBid molecules which can be labeled by the free MTSSL.

The mitochondria were then separated from the soluble fraction by centrifugation, and room temperature EPR spectra were detected both in the pellet resuspended in 10 µl of buffer and in the supernatant. Spin concentration was determined to compare the cBid_R1_ amount in all fractions. Notably, the membrane bound portion of cBid_R1_ was decreased when unlabeled cBid was added (Fig. 7). The sample containing the competitor unlabeled cBid in solution showed a reproducible reduction in the amount of spins bound to the mitochondria, indicating that the bound fraction is in a dynamic equilibrium with the soluble fraction (see also Fig. S4). Taking into account the possible occurrence of an auto-spin-labeling of cBid from the label released in the sample, the reduction in the bound spins observed at the mitochondria confirms that the bound cBid molecules can be exchanged by the unlabeled moieties in solution. Analogously to what found for liposomes, only a fraction of the total cBid population interacts with the mitochondria, as can be seen from the SDS-PAGE performed on the pellet and on the supernatant fractions after incubation with mitochondria (Fig. S4). Interestingly, we found that cBid has a stronger tendency to interact with mitochondria than with liposomes (Fig. S5), suggesting a possible role of specific mitochondrial proteins (e.g. [37], [38]).

The data obtained in mitochondria by EPR provide information which cannot be obtained by conventional biochemical techniques, and validate the idea that cBid molecules repeatedly exchange between their membrane bound and soluble conformation, and contradict the hypothesis that once bound, cBid stays permanently at the membrane. Moreover, our experiments show that cBid binds to liposomes, but to a greater extent to mitochondria, highlighting the role of mitochondrial proteins in the binding events.

## Discussion

Cleavage by Caspase-8 triggers the release of the p15 fragment (tBid) from cBid which in turns activates Bax at the membrane bilayer. Caspase-8 cleavage alone was previously shown by NMR not to induce protein dissociation at millimolar concentrations [23]. Here we make use of the two spin labeled natural cysteines, one in p15 and one in p7 to monitor the dissociation events by DEER at lower protein concentration. The EPR data confirm that no dissociation occurs in cBid in the micromolar concentration range. The overall conformation of Bid before and after cleavage is maintained, as proven by the invariant 2.1 nm distance between the spin labels attached at cysteines 126 and 30. On the other hand, cleavage induced an alteration in the melting temperature of cBid, which correlates with decreased stability. Complete removal of p7 destabilizes p15 in solution shown as lower melting temperature and strong tendency to aggregate. Together, these findings indicate that Caspase-8 cleavage can only prime Bid for fragment dissociation, without inducing relevant conformational changes in the protein.

The full dissociation of p15 from p7 in cBid was obtained only in the presence of detergents, and this dissociation is shown here by EPR to be fully reversible. Fluorescence methods validated the reversibility even at submicromolar concentration, indicating that the remarkably strong and specific interaction between the two protein fragments is a unique feature of cBid. Detergents are thought to destabilize the hydrophobic interface between p15 and p7, inducing the exposure of the hydrophobic protein core in full length Bid and dissociation of the two polypeptides in the cleaved variant. Interestingly, the hydrophobic interactions holding p7 and p15 together are strong enough to resist treatment with detergent micelles up to a certain concentration, and only high detergent concentration could lead to a complete complex dissociation. If fragment dissociation is a required step for Bax activation, the specific p15-p7 interactions might allow a series of complex dissociation and re-association events, which could possibly enable repetitive activation of several Bax proteins by the same Bid molecule, in line with the proposed “hit-and-run" model of Bid-induced Bax activation.

From experiments performed on isolated mitochondria, we found that cBid switches repeatedly between a soluble and a membrane-bound conformation. This is in line with our previous hypothesis that cBid catalyzes Bax membrane inserting [13].

Our results demonstrate that Bid reacts quite differently to the addition of detergents compared to Bax: Bax undergoes a conformational change triggering oligomer formation, whilst the hydrophobic core of FL-Bid is exposed to the solvent and cBid completely dissociates.

Moreover, Bid was found to be unable to build oligomers, either in the presence of detergents or in liposomes. Only the isolated tBid was shown to be prone to dimerization (via disulfide bridge formation) in the absence of reducing agents and the tBid dimers retained the ability to activate Bax (data not shown). However, in the cell tBid is probably monomeric, nonetheless tBid dimers were identified at the mitochondrial membrane [39] and the altered redox potential of a cell during apoptosis could possibly enable disulfide bridge formation (e.g. [40], [41]).

Again in contrast to Bax, which is completely membrane inserted in the oligomeric form [13], addition of liposomes induced fragment dissociation in a small fraction of cBid and membrane binding of p15 (tBid). ECL and MOML liposomes induced only partial cBid disassembly and the p15 fragment was found in the membrane fraction, but the majority of the complexes did not change conformation and stayed in solution. Interestingly, the minor cBid disassembly causing p15 to interact with the membrane occurred immediately after liposomes addition (within 2 minutes) and the level of complex dissociation or membrane binding was not increasing over time. cBid showed a higher propensity to bind to mitochondria than to liposomes. Two factors can be important for cBid binding to membranes: one being a specific type of lipids (e.g. cardiolipin [42]), the other being a specific protein, as for example MTCH2/MIMP, recently found to facilitate tBid recruitment at the MOM [37]. The number of binding factors for cBid in the membrane determines the amount of bound cBid and interestingly mitochondria could bind more cBid than liposomes at similar lipid concentration (Fig. S5).

The exchange we found between membrane bound and soluble proteins implies that dissociation and association events are in equilibrium and cBid cycles between the solution and the membrane, or in other words that the binding of cBid to the membrane is transient. We suggest that this cycling has a functional significance, enabling Bid to act as a catalytic trigger or as a “chaperone" for the membrane insertion of other Bcl-2 proteins. In fact, the reversible disassembly and re-assembly of cBid would allow this assistance to occur repeatedly, and would explain why Bid completely activates Bax at stoichiometric ratios much less then 1∶1 [13], [14]. In contrast to cBid, tBid was found mainly membrane-associated. Notably, our experiments demonstrate clearly that p7 needs to be actively removed from the system (e.g. by degradation [17]), otherwise p15 and p7 are very likely to re-associate to form the complex.

Interestingly, a recent publication also showed a Bcl-xL dependent retranslocation of Bax from the outer mitochondrial membrane to the cytosol [43]. This, together with the data presented here, suggests that the interplay between the different Bcl-2 proteins and the mitochondrial membrane is even more complex than expected. More studies will be necessary to understand the complex interaction of all the players, to enable us in the future to manipulate these networks by drugs to desensitize cancer cells for apoptosis.

This study establishes an experimental setup to measure spin labeled proteins on mitochondria, a first step towards *in vivo* EPR for apoptotic proteins and highlights the very different modes of action of the two Bcl-2 proteins Bid and Bax. Bid is shown to be an extremely adaptable protein able to switch between soluble and membrane bound as well as open and closed conformations. This intrinsic adaptability may allow Bid to interact with Bax as well as with its counter-player Bcl-xL. Studies are in progress to delineate the conformational changes of the Bcl-2 proteins when the three active players Bid, Bax and Bcl-xL meet at the mitochondrial membrane.

## Materials and Methods

### Expression and purification of Bid

Mouse Bid was expressed and purified according to [44] and [13]. Mouse Bid has one natural cysteine in p15 and one in p7, thus it was more suitable for EPR studies than the human Bid protein containing three cysteines in the N-terminal cleaved fragment. The plasmid containing the sequence for the Bid mutant was produced by site-specific mutagenesis of pET23-mouse_Bid. The protein fragments obtained (cBid) were further purified by Ni-affinity chromatography and analyzed by SDS- PAGE and LC-MS (micrOTOF LC, Bruker Daltonics, Billerica, MA). Bid was shown to be exclusively cleaved before Gly 60 (Gly 68 in the His-tagged protein). tBid was eluted with buffer A (20 mM Tris, 150 mM NaCl, pH 7.5) containing 2% octyl-glucoside (Anatrace Inc, Maumee, OH). The detergent was afterwards removed by dialysis.

### Isolation of mitochondria

Liver mitochondria were isolated from albino Wistar rats (150–180 g) by differential centrifugation from the liver following [45]. The liver was quickly removed and was homogenized in a Potter-Evelhjem homogenizer with isolation medium containing 250 mM sucrose, 1 mM Tris-HCl, pH 7.4 at 4°C. The homogenate was centrifuged at 1100×g for 10 min and the resulting supernatant was centrifuged at 5000×g for 10 min. The obtained pellet was washed by resuspending in the same buffer, and centrifuged at 12520×g for 10 min to obtain the mitochondrial pellet. Mitochondrial protein was assayed by the Biuret method. Quantitative phosphorus analysis to obtain the lipid content was performed according to [46]. The lipid concentration was determined to be 10 mg/ml. The mitochondria suspension was diluted with buffer A to reach 1.6 mg/ml final lipid concentration (5 mg/ml protein concentration) and incubated with cBid_R1_ (20 µM).

### Spin labeling of Bid

The MTSSL spin label (1-oxyl-2,2,5,5-tetramethlpyrroline-3-methyl methanethiosulfonate, TRC, Toronto, Canada) was covalently attached to the cysteine residues of the monomeric Bid in water solution by overnight incubation at 4°C with 10-fold MTSSL molar excess in buffer A. Excess of label was removed by extensive dialysis. For tBid, a 2-fold MTSSL excess was used and the unbound label was only partially removed due to the tendency of tBid to aggregate.

### Detergents and lipids

Detergents were purchased from Anatrace and lipids from Avanti polar lipids. The *E. coli* polar lipid mixture (20 mg/ml) in buffer A was used to prepare liposomes (ECL). Liposomes mimicking the mitochondrial outer membrane composition (MOML) were prepared as in [14] with 18.4 mg egg PC L-α-phosphatidylcholine, 10 mg egg PE L-α phosphatidylethanolamine, 4.4 mg bovine liver PI L-α-phosphatidylinositol, 4.0 mg 18∶1 phosphatidylserine, 3.2 mg cardiolipin hydrated in 2 ml of buffer A. Both liposomes were extruded at 400 nm prior to the measurements.

### Size exclusion chromatography

All size exclusion chromatography experiments were performed on a Superdex 75 column using a SMART chromatography system (GE Healthcare Bio-Sciences AB, Uppsala, Sweden).

### Circular-dichroism spectroscopy and secondary structure determination

The CD-spectra and melting curves were recorded on a Jasco J715 spectropolarimeter (Jasco, Gross Umstadt, Germany) with a Jasco PFD 350S Peltier type FDCD attachment for temperature control using a 0.1 mm quartz cuvette. Two spectra were accumulated per measurement using a data pitch of 0.1 nm, a scan speed of 20 nm s^−1^ and 1 nm slit width. The concentration of Bid was adjusted to 0.1–0.2 mg/ml. The content of secondary structure was calculated using the program CDNN [47].

### SDS PAGE

Commercially available gels und buffers were used as described by the manufacturer (Invitrogen).

### EPR spectroscopy

Room temperature continuous wave (cw) electron paramagnetic resonance (EPR) spectra were recorded on an Elexsys E500 X-band spectrometer equipped with a super high Q Bruker cavity, with 0.6 mW microwave power and 0.15 mT B-field modulation amplitude. Protein concentrations for EPR measurements were in the 40–180 micromolar range. For pulse EPR experiments samples were shock frozen in liquid nitrogen in quartz EPR tubes (3 mm outer diameter) in the presence of 30% v/v deuterated glycerol. Dipolar time evolution data were recorded at 50 K with a dead-time free four pulse sequence (double electron electron resonance, DEER [48]) at X-band frequencies with a Bruker Elexsys 580 spectrometer equipped with a Bruker Flexline split-ring resonator ER 4118X-MS3 and a continuous flow He cryostat ESR900 (Oxford Instruments, Abingdon, Oxfordshire, UK). All observer pulses were set to 32 ns and the pump pulse to 12 ns. Deuterium nuclear frequencies were averaged. Data analysis was performed with the software DeerAnalysis2010 [49]. Continuous wave spectra and DEER traces were reproduced at least three times on different protein batches.

### Fluorescence spectroscopy

Fluorescence emission spectra were recorded on Bid derivatives (0.5 µM) on a Perkin-Elmer spectrometer (LS50B, Waltham MA) with excitation at 280 nm (5 nm slit width).

### Confocal microscopy

cBid was labeled with Alexa Fluor® 633 C5 maleimide (Invitrogen). GUVs containing 20% cardiolipin, 80% Egg-PC (mol/mol) and <0.05% DiO were formed by electroformation [50] and observed at room temperature in multitrack mode on a LSM710 confocal fluorescence microscope using a C-Apochromat 40× 1.2 water immersion objective (Zeiss, Jena, Germany). For the first track, the excitation light was from an Ar-ion laser (488 nm) and a spectral beam guide was used to detect between 494 and 554 nm. For the second track, the sample was excited with a He-Ne laser at 633 nm and detected between 638 and 747 nm. Images were processed with ImageJ (http://rsbweb.nih.gov/ij/).

## Supporting Information

Figure S1 **Comparison of the CD spectra of Bid variants.** Normalized CD spectra of FL-Bid, cBid and tBid in the absence and presence of detergent. Black and red, soluble and detergent-incubated (1% DDM) FL-Bid, respectively; gray and orange, soluble and detergent-incubated (1% DDM) cBid, respectively; blue, soluble tBid.
(TIF)

Figure S2 **Reproducibility of the spectral features.** A. Room temperature continuous wave EPR spectra of three different batches of spin-labeled cBid. B. DEER form factors and distance distributions obtained on two different batches of spin-labeled cBid (black and grey traces).
(TIF)

Figure S3 **Effect of detergents on singly labeled Bid.** A. Room temperature continuous wave EPR spectra of FL-Bid_R1_ (upper panel, grey) and FL-Bid_C126R1_ (upper panel, black). Spectra of the singly labeled Bid moiety carrying the spin label at position 126 in the presence of 2% DDM and 2% OG are presented in red and cyan, respectively. Arrows highlight the immobile (i) and mobile (m) spectral components. (B) Analogous room temperature spectra of the cleaved variants. B. Room temperature continuous wave EPR spectra of cBid_R1_ and cBid_C126R1_ under the same conditions.
(TIF)

Figure S4 **Reproducibility of the results obtained with mitochondria at lower protein concentrations.** A. Room temperature continuous wave EPR spectra of cBid_R1_ in the presence of isolated mitochondria (2 mg/ml lipid concentration). The central upper spectrum of cBid_R1_ is obtained immediately after addition of mitochondria. The left column shows the control spectrum after 1 h incubation at 37°C (addition of 15 µl buffer after the first 20 min incubation, spin concentration 13 µM); the spectrum of the pellet resuspended in 10 µl buffer (calculated spin concentration 6.5 µM) and the spectrum in the supernatant. The right column shows the analogous spectra obtained after addition of 20-fold protein excess of competitor unlabeled cBid (addition of 15 µl of cBid 800 µM after the first 20 min incubation, spin concentration 12.5 µM). The spectrum in the pellet (spin concentration 3.5 µM) reveals the reduced amount of spin labeled protein at the membrane in the presence of competitor unlabeled protein. B. Zoom in a SDS-PAGE gel showing the comparison between the supernatant and pellet fractions used for the EPR experiments in the absence and presence of competitor. The fraction of non interacting p15 is visible in the supernatant fractions. In the pellet fractions a clear sign of p15 is visible only in the sample containing the excess of competitor unlabeled cBid.
(TIF)

Figure S5 **Comparison between mitochondria and** ***E. coli*** **liposomes.** The upper central spectrum of cBidR1 is obtained immediately after addition of mitochondria (60 µl sample, calculated spin concentration 42 µM). Left, room temperature continuous wave EPR spectra of cBidR1 in the presence of isolated mitochondria (1.6 mg/ml lipid concentration) as in Fig. 7. The left column shows the control spectrum after 1 h incubation at 37°C (addition of 20 µl buffer after the first 20 min incubation, spin concentration 32 µM); the spectrum of the pellet resuspended in 10 µl buffer (calculated spin concentration 36 µM) and the spectrum in the supernatant (calculated spin concentration 21 µM). Right, analogous spectra obtained in the presence of freshly extruded liposomes formed with *E. coli* polar lipid extract (5 mg/ml lipid concentration). The spin concentration calculated for all other fractions are presented in the figure. The pellet fraction contains about 3 times less cBidR1 than the mitochondrial counterpart, suggesting a higher tendency of cBid to interact with the mitochondria than with the liposomes.
(TIF)
