# Supplementary figures and images for: Dynamic Interaction of cBid with Detergents, Liposomes and Mitochondria
Source: PLoS One. 2012 Apr 23;7(4):e35910. doi: 10.1371/journal.pone.0035910 (PMC3335097; doi:10.1371/journal.pone.0035910)

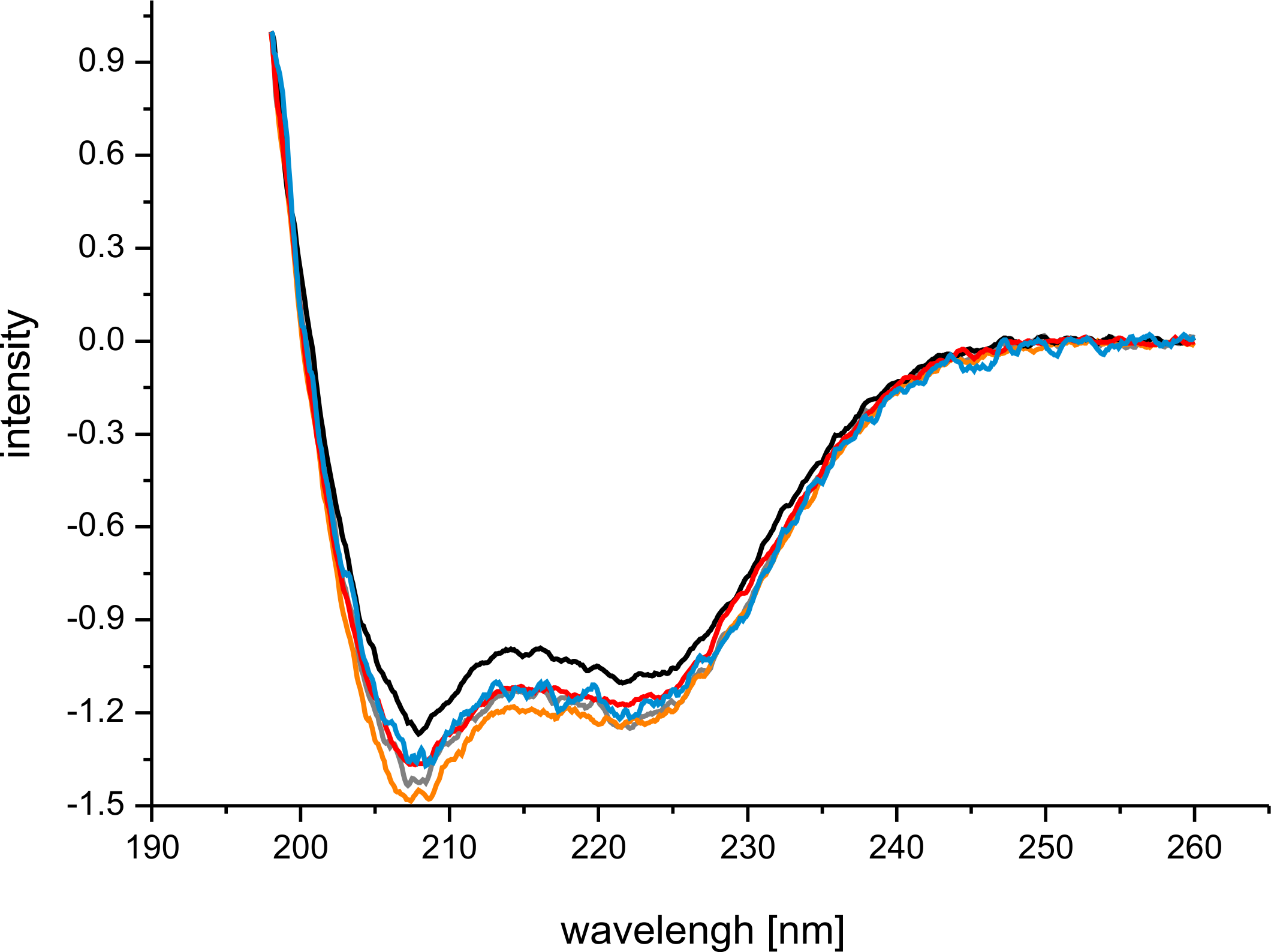

Supplement: Figure S1 — Comparison of the CD spectra of Bid variants. Normalized CD spectra of FL-Bid, cBid and tBid in the absence and presence of detergent. Black and red, soluble and detergent-incubated (1% DDM) FL-Bid, respectively; gray and orange, soluble and detergent-incubated (1% DDM) cBid, respectively; blue, soluble tBid. (TIF) [file pone.0035910.s001.tif]

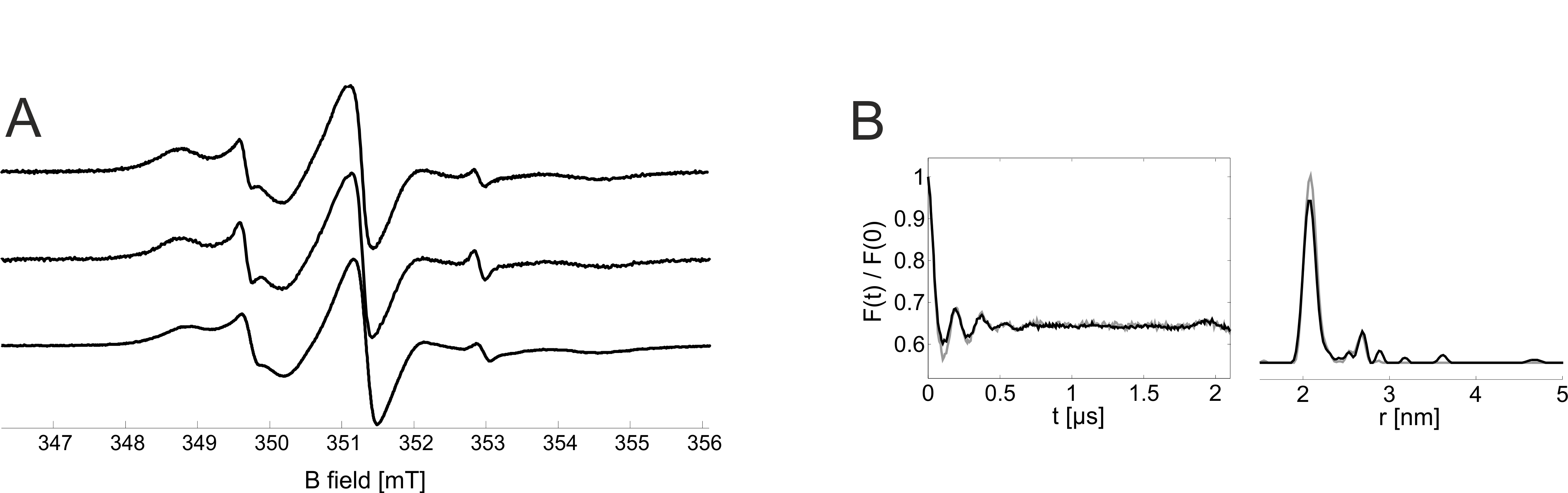

Supplement: Figure S2 — Reproducibility of the spectral features. A. Room temperature continuous wave EPR spectra of three different batches of spin-labeled cBid. B. DEER form factors and distance distributions obtained on two different batches of spin-labeled cBid (black and grey traces). (TIF) [file pone.0035910.s002.tif]

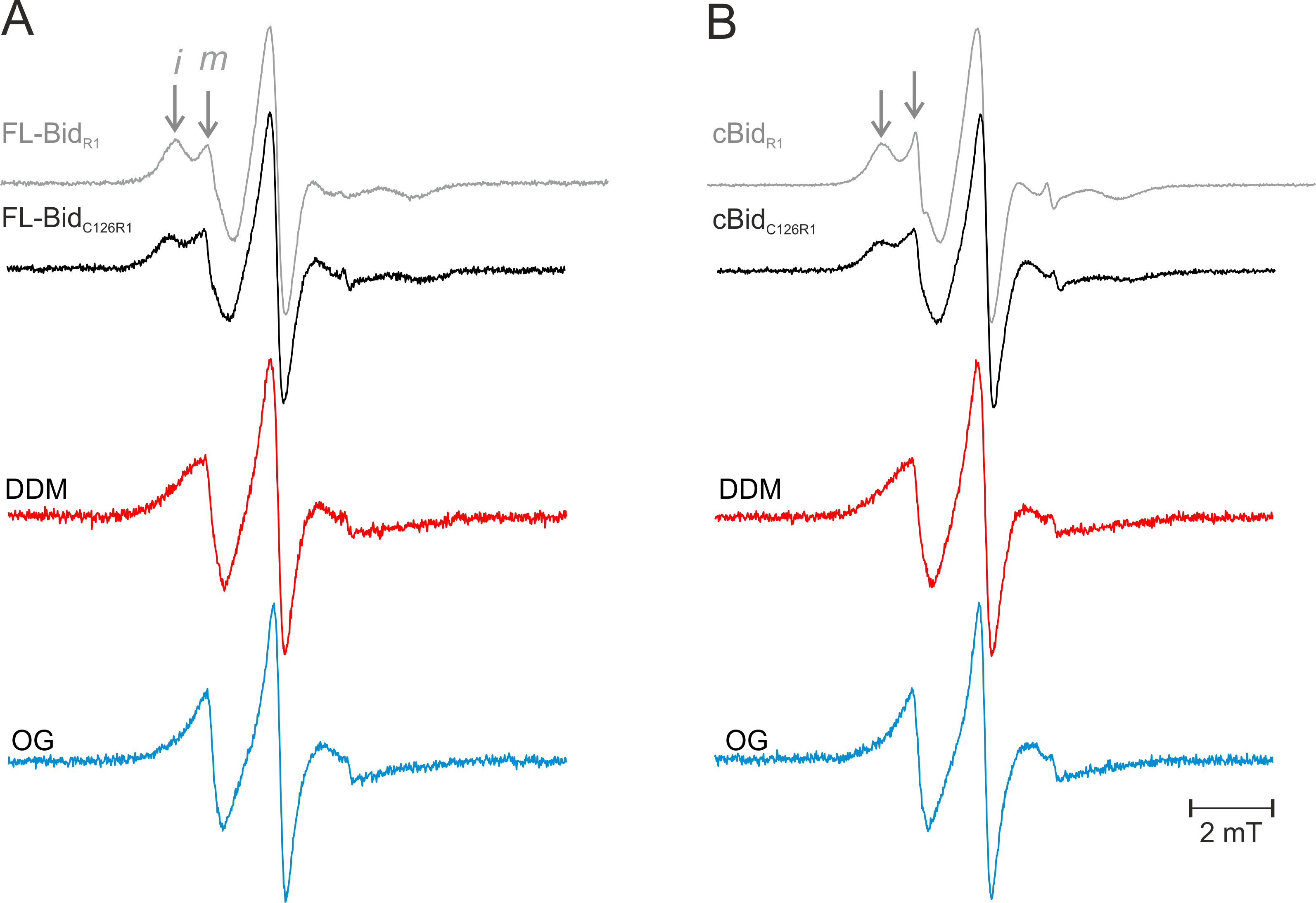

Supplement: Figure S3 — Effect of detergents on singly labeled Bid. A. Room temperature continuous wave EPR spectra of FL-BidR1 (upper panel, grey) and FL-BidC126R1 (upper panel, black). Spectra of the singly labeled Bid moiety carrying the spin label at position 126 in the presence of 2% DDM and 2% OG are presented in red and cyan, respectively. Arrows highlight the immobile (i) and mobile (m) spectral components. (B) Analogous room temperature spectra of the cleaved variants. B. Room temperature continuous wave EPR spectra of cBidR1 and cBidC126R1 under the same conditions. (TIF) [file pone.0035910.s003.tif]

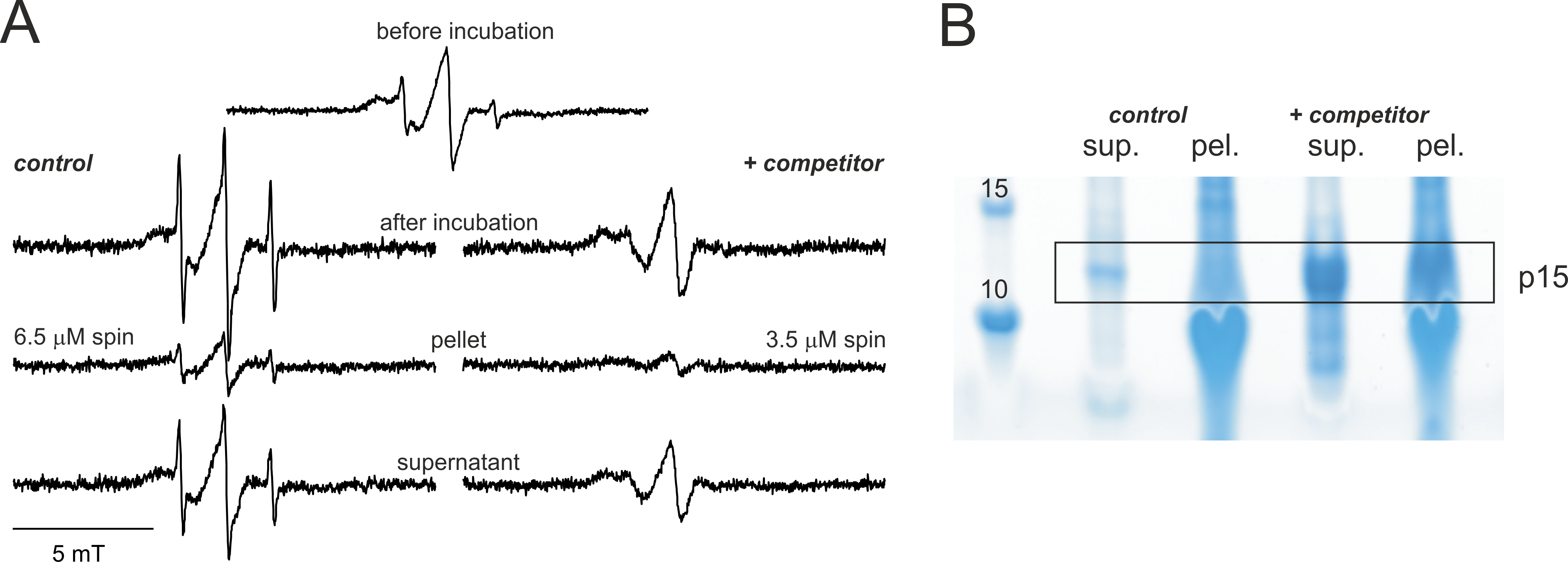

Supplement: Figure S4 — Reproducibility of the results obtained with mitochondria at lower protein concentrations. A. Room temperature continuous wave EPR spectra of cBidR1 in the presence of isolated mitochondria (2 mg/ml lipid concentration). The central upper spectrum of cBidR1 is obtained immediately after addition of mitochondria. The left column shows the control spectrum after 1 h incubation at 37°C (addition of 15 µl buffer after the first 20 min incubation, spin concentration 13 µM); the spectrum of the pellet resuspended in 10 µl buffer (calculated spin concentration 6.5 µM) and the spectrum in the supernatant. The right column shows the analogous spectra obtained after addition of 20-fold protein excess of competitor unlabeled cBid (addition of 15 µl of cBid 800 µM after the first 20 min incubation, spin concentration 12.5 µM). The spectrum in the pellet (spin concentration 3.5 µM) reveals the reduced amount of spin labeled protein at the membrane in the presence of competitor unlabeled protein. B. Zoom in a SDS-PAGE gel showing the comparison between the supernatant and pellet fractions used for the EPR experiments in the absence and presence of competitor. The fraction of non interacting p15 is visible in the supernatant fractions. In the pellet fractions a clear sign of p15 is visible only in the sample containing the excess of competitor unlabeled cBid. (TIF) [file pone.0035910.s004.tif]

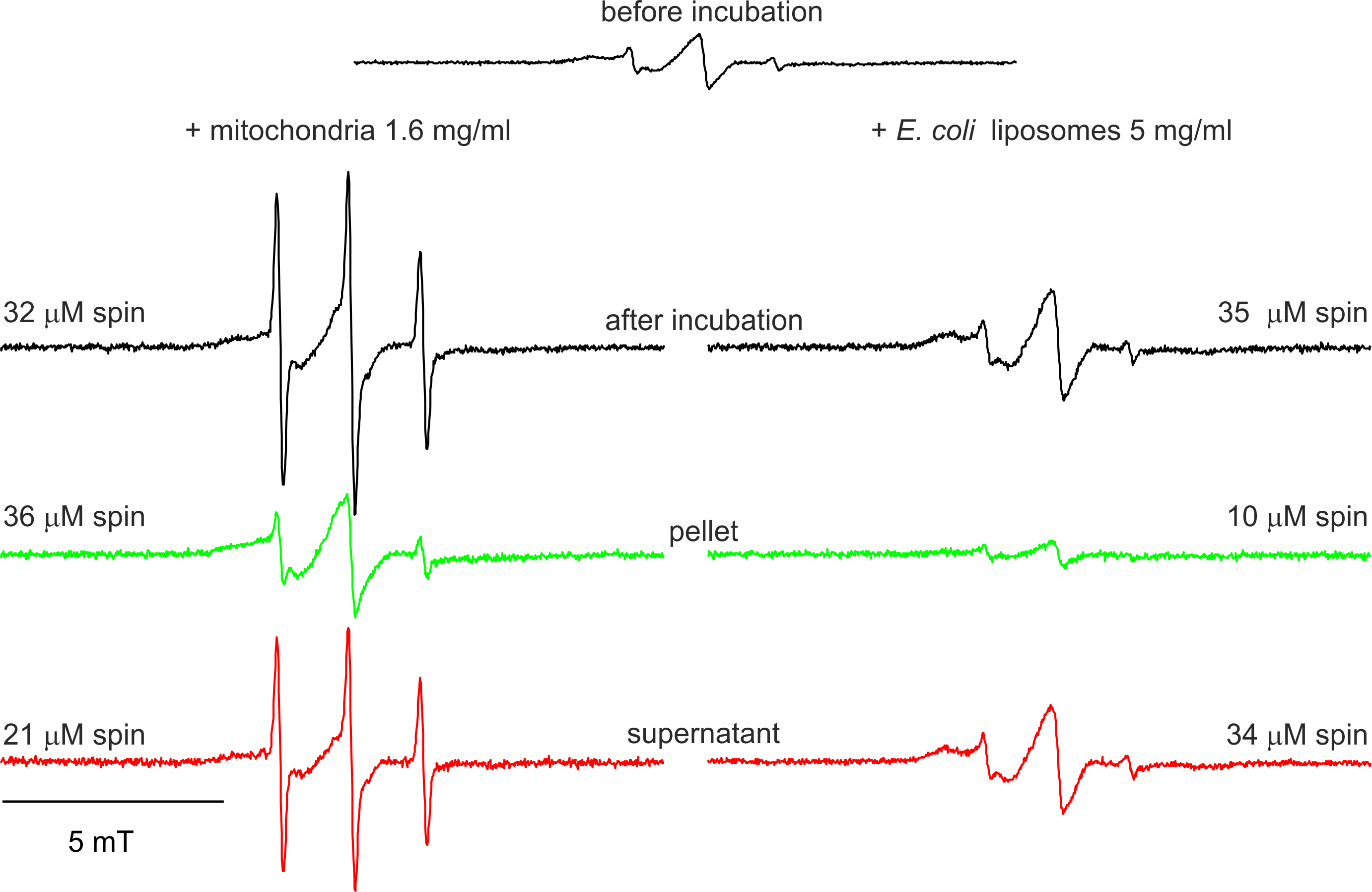

Supplement: Figure S5 — Comparison between mitochondria and E. coli liposomes. The upper central spectrum of cBidR1 is obtained immediately after addition of mitochondria (60 µl sample, calculated spin concentration 42 µM). Left, room temperature continuous wave EPR spectra of cBidR1 in the presence of isolated mitochondria (1.6 mg/ml lipid concentration) as in Fig. 7. The left column shows the control spectrum after 1 h incubation at 37°C (addition of 20 µl buffer after the first 20 min incubation, spin concentration 32 µM); the spectrum of the pellet resuspended in 10 µl buffer (calculated spin concentration 36 µM) and the spectrum in the supernatant (calculated spin concentration 21 µM). Right, analogous spectra obtained in the presence of freshly extruded liposomes formed with E. coli polar lipid extract (5 mg/ml lipid concentration). The spin concentration calculated for all other fractions are presented in the figure. The pellet fraction contains about 3 times less cBidR1 than the mitochondrial counterpart, suggesting a higher tendency of cBid to interact with the mitochondria than with the liposomes. (TIF) [file pone.0035910.s005.tif]
